# Effects of different supplements on Hashimoto’s thyroiditis: a systematic review and network meta-analysis

**DOI:** 10.3389/fendo.2024.1445878

**Published:** 2024-12-04

**Authors:** Bingcong Peng, Weiwei Wang, Qingling Gu, Ping Wang, Weiping Teng, Zhongyan Shan

**Affiliations:** Department of Endocrinology and Metabolism, The Institute of Endocrinology, National Health Commision of the People's Repiublic of China (NHC) Key Laboratory of Diagnosis and Treatment of Thyroid Diseases, The First Affiliated Hospital of China Medical University, Shenyang, China

**Keywords:** vitamin D, selenium, myo-inositol, Hashimoto’s thyroiditis, meta-analysis

## Abstract

Clinicians often consider the use of dietary supplements to assist in lowering thyroid autoantibody titres in patients with Hashimoto’s thyroiditis (HT). Currently, different supplements differ in their ability to reduce autoantibody levels. The purpose of this article is to compare the ability of different supplements to lower autoantibody titres and restore TSH levels through a systematic literature review. We obtained information from the PubMed, Web of Science, Embase, and Cochrane databases, as well as the China National Knowledge Infrastructure (CNKI). Selected studies included those using selenium, Vitamin D, Myo-inositol, and Myo-inositol in combination with selenium for the treatment of HT patients with euthyroidism. These data were combined using standardised mean differences (SMDs) and assessed using a random effects model. A total of 10 quantitative meta-analyses of case-control studies were selected for this meta-analysis. Compared to the placebo group, the use of selenium supplements was able to significantly reduce the levels of thyroid peroxidase autoantibodies (TPOAb) (SMD: -2.44, 95% CI: -4.19, -0.69) and thyroglobulin autoantibodies (TgAb) (SMD: -2.76, 95% CI: -4.50, -1.02). During a 6-month treatment, the use of Myo-inositol, Vitamin D alone, and the combination of selenium, and Myo-inositol did not effectively reduce TPOAb (Myo-inositol: SMD:-1.94, 95% CI: -6.75, 2.87; Vitamin D: SMD: -2.54, 95% CI: -6.51,1.42; Se+Myo-inositol: SMD: -3.01, 95% CI: -8.96,2.93) or TgAb (Myo-inositol: SMD:-2.02, 95% CI: -6.52, 2.48; Vitamin D: SMD: -2.73, 95% CI: -6.44,0.98; Se+Myo-inositol: SMD: -3.64, 95% CI: -9.20,1.92) levels. Therefore, we recommend that patients with HT(Hashimoto’s Thyroiditis) be given an appropriate amount of selenium as an auxiliary treatment during standard-of-care treatment.

## 1 Introduction

Hashimoto’s thyroiditis(HT) is a common autoimmune disease with a prevalence of 14.19% in China and is 2-3 times more common in women than in men (1).HT is typically characterised by euthyroidism (2). The development of HT is associated with genetic susceptibility (3), iodine intake (4), and other factors. Its pathology is characterised by thyroid cell destruction and lymphocytic infiltration (5). On examination, patients usually present with a significant increase in thyroid peroxidase antibodies (TPOAb) or thyroglobulin antibodies (TgAb), with normal or elevated levels of TSH(Thyroid stimulating hormone) (6). Clinically, levothyroxine is used to manage the symptoms of hypothyroidism (7). However, studies have demonstrated that TPOAb are linked to unfavourable pregnancy outcomes (8) and lower IQ(intelligence quotient) in children (9). Consequently, the use of supplements to reduce TPOAb or TgAb levels is also being considered by clinicians for treatment of HT (10–12).

The highest concentration of selenium across tissues is found in the thyroid (13). Selenium plays a variety of roles in the thyroid, the most important of which is its involvement in the formation of type I deiodinase, which catalyses the deiodination of tetraiodothyronine (T4) to triiodothyronine (T3) (14). A deficiency of delenium has demonstrated to reduce the activity of deiodinase, which consequently alters thyroid hormone metabolism, as evidenced by the blockage of T4 to T3 conversion (15). In an epidemiological study of selenium and goitre in China, the prevalence of pathological thyroid disease was significantly higher in selenium-deficient areas than in selenium-sufficient areas (16), indicating that adequate selenium supplementation may be beneficial in the treatment of HT, in addition to its known anti-inflammatory effects (17–20).

Vitamin D plays a significant role in maintaining calcium and phosphorus balance and bone health (21, 22). There is a bidirectional relationship between human vitamin D levels and the thyroid function (23, 24). Furthermore, numerous studies have documented a negative correlation between antithyroid antibodies and 25(OH)D levels (25–27). Conversely, HT has been demonstrated to impact bone metabolism (28). Many meta-analyses and clinical trials have shown that supplementation with moderate amounts of vitamin D is beneficial in reducing autoantibody levels in patients with HT (29). However, some studies have not yielded comparable outcomes (30).

In addition to selenium and Vitamin D supplementation in HT patients, Krysiak et al. recently used selenium and Vitamin D in combination with inositol (35). This combination was effective in lowering autoantibody levels, but due to the reduced sample size of the study, these findings warrant validation across larger patient cohorts.

In this study, the effects of supplementation with selenium, Vitamin D, and inositol, or a combination of these, in patients with HT is summarised. A systematic review and network meta-analysis was performed to assess the effects of these supplements on TPOAb, TgAb, and TSH, based on recently published randomised controlled trials.

## 2 Materials and methods

### 2.1 Search strategy

A systematic literature search was performed using the PubMed, Web of Science, Embase, and Cochrane databases, as well as the China National Knowledge Infrastructure (CNKI) in December 2023 using the following search terms: (Hashimoto Disease[MeSH Terms]) OR (Disease, Hashimoto[Other Term]) OR (Chronic Lymphocytic Thyroiditis[Other Term]) OR (Chronic Lymphocytic Thyroiditides[Other Term]) OR (Lymphocytic Thyroiditides, Chronic[Other Term]) OR (Lymphocytic Thyroiditis, Chronic[Other Term]) OR (Thyroiditides, Chronic Lymphocytic[Other Term]) OR (Thyroiditis, Chronic Lymphocytic[Other Term]) OR (Hashimoto Struma[Other Term]) OR (Hashimoto’s Struma[Other Term]) OR (Hashimoto’s Syndrome[Other Term]) OR (Hashimoto Syndrome[Other Term]) OR (Hashimoto’s Syndromes[Other Term]) OR (Hashimotos Syndrome[Other Term]) OR (Syndrome, Hashimoto’s[Other Term]) OR (Syndromes, Hashimoto’s[Other Term]) OR (Hashimoto’s Disease[Other Term]) OR (Disease, Hashimoto’s[Other Term]) OR (Hashimotos Disease[Other Term]) OR (Hashimoto Thyroiditis[Other Term]) OR (Hashimoto Thyroiditides[Other Term]) OR (Thyroiditides, Hashimoto[Other Term]) OR (Thyroiditis, Hashimoto[Other Term]) AND (selenium[MeSH Terms]) OR (Vitamin d[MeSH Terms]) OR (Myo-inositol[MeSH Terms]).

### 2.2 Study selection

Relevant literature was selected in PubMed, Cochrane Central Register of trials, and Web of Science. The search terms were in English, and the article language was restricted to English. The words or technical terms used for the search were related to “Autoimmune thyroiditis”, “Hashimoto’s thyroiditis”, “Selenium”, “Vitamin D”, and “Myo-inositol”.

Studies included in this meta-analysis had to meet the following criteria: (I)Participants were diagnosed with HT; (II) Patients enrolled in the study did not use levothyroxine sodium tablets throughout the study; (III) Patients did not suffer from other autoimmune or metabolic disease; (IV) Patients in experimental and control groups did not take any supplements within six months (particularly inositol, Vitamin D, and selenium); (V) Patients with increased TPOAb or TgAb titres; (VII) Patients did not suffer from congestive heart failure, diabetes, renal or hepatic impairment, and were not pregnant or lactating.

Wang Ping and Gu Qing Ling reviewed and quality-assessed each article’s title, abstract, and/or full text retrieved from the literature search to determine eligibility for the meta-analysis.

### 2.3 Data extraction and quality assessment

Data extraction was performed using an extraction table that focussed on study characteristics (first author, year of publication, and study design), participant characteristics (number, subgroup, age), outcomes (levels of TPOAb, TgAb, and TSH), and adjusted matching factors.

### 2.4 Quality assessment

Two reviewers completed quality assessment of the selected studies using the Jadad scale. This included assessment of whether the studies were blinded, and randomised, and whether subjects withdrew from the study or were lost to follow-up. The total score ranged from 3 to 5, with higher scores representing a higher assessment quality. All discrepancies or conflicting assessments were resolved via a consensus discussion with a third reviewer.

### 2.5 Statistical analysis

Mean differences with a 95% CI(Confidential intervals) were used to evaluate the impact of each supplement on HT-related and representative metrics. To account for the heterogeneity among studies, the data were pooled using a random-effects method. Heterogeneity was assessed using the I² statistic, and I² > 50% was considered a level of high heterogeneity. Subgroup analysis was performed according to the time and dose of the supplements used. All analyses were performed using STATA (version 17.0, StataCorp), and a P<0.05 was considered statistically significant.

## 3 Results

### 3.1 Characteristics of the included articles

As illustrated in **Table 1**, the literature search yielded a total of 798 relevant articles. Following a comprehensive examination of the article’s abstracts, 193 studies with full-text publications were subjected to a detailed evaluation. Of these, 184 studies were excluded due to the presence of irrelevant or single-arm results, leaving a total of 10 articles for inclusion in the meta-analysis. **Table 2** presents the abstract items for these 10 articles, including the first author, publication year, study design, sample size, results, adjusted matching factors, and quality assessment scores. **Table 3** shows the supplements' dosage and changes in TPOAb, TgAb and TSH before and after supplementation.

**Table 1:** Flowchart of the search strategy and study selection process for this meta-analysis.

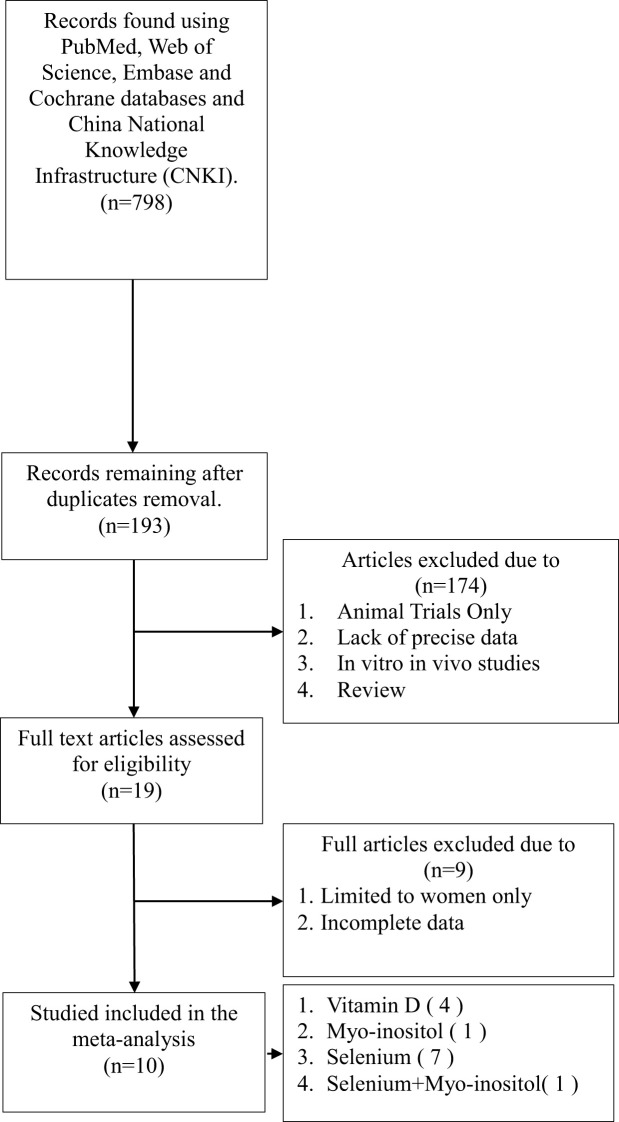

**Table 2:** Characteristics of the studies included in the meta-analysis.

Study	Country	Intervention	Study Group	Age (y)	Control Group	Age (y)	Treatment Time(months)	Quality
M.NORDIO 2017 (32)	Italy	Selenium+Myo-inositol;Placebo	84	NM	84	NM	6	5
Robert Krysiak 2023 (31)	Poland	Vitamin D,Selenomethionine,Myo-inositol	VD:29 Se:29 MI:29	VD:32 ± 7; Se:32 ± 6; MI:31 ± 6	29	31 ± 7	6	3
Robert Krysiak 2018-1 (33)	Poland	Vitamin D;Selenomethyionine(Control group)	20	35 ± 8	17	34 ± 7	6	6
Robert Krysiak 2018-2 (34)	Poland	Selenium	23	32 ± 6	24	31 ± 6	6	6
Qu Chunmei 2018 (35)	China (CNKI)	Selenium	60	NM	60	NM	6	4
Lv Xiaofei 2018 (36)	China (CNKI)	Selenium	40	28.8 ± 2.2	40	28.1 ± 2.3	6	6
Han Na 2018 (37)	China (CNKI)	Selenium	30	43.7 ± 10.1	30	42.6 ± 11	6	6
Gao Hongxia 2017 (38)	China (CNKI)	Selenium	46	NM	44	NM	3	4
Zhou Jingying 2018 (39)	China (CNKI)	Vitamin D	61	NM	61	NM	3	4
Fu Liping 2019 (40)	China (CNKI)	Vitamin D	36	49.56 ± 7.88	34	57.11 ± 11.42	3	7

**Table 3:** Clinical parameters and microbiology assessment of selected studies.

Study	Dosage of Intervention	Pre-treatment TPOAb(IU/mL)	Post-treatment TPOAb(IU/mL)	Pre-treatment TgAb(IU/mL)	Post-treatment TgAb(IU/mL)	Pre-treatment TSH(μIU/ml)	Post-treatment TSH(μIU/ml)
M.NORDIO 2017 (32)	Se:16.6mg(83ug) MI(600mg)+Se(16.6mg)	MI+Se: 733.7 ± 485.8 Se:820.13 ± 513.99	MI+Se: 614.4 ± 472 Se:724.51 ± 524.98	MI+Se:355 ± 220.5 Se:415.43 ± 275.89	MI+Se:298.8 ± 216.6 Se:422.03 ± 256.74	MI+Se:4.22 ± 0.6 Se:4.32 ± 0.86	MI+Se:3.26 ± 0.89 Se:4.23 ± 0.89
Robert Krysiak 2023 (31)	Vitamin D:Vitamin D:100μg(4000IU);Selenomethionine:200μg/d,Myo-inositol:2.0g/d	Vitamin D: 912 ± 365 Se: 895 ± 279 MI:928 ± 370 Placebo:882 ± 324	Vitamin D:551 ± 290 Se:680 ± 221 MI:712 ± 286 Placebo:905 ± 348	Vitamin D:832 ± 354 Se:861 ± 324 MI:870 ± 280 Placebo:845 ± 382	Vitamin D:535 ± 267 Se:623 ± 265 MI:712 ± 286 Placebo:882 ± 364	Vitamin D:2.9 ± 0.8 Se:3.1 ± 0.7 MI:3.0 ± 0.6 Placebo:3.0 ± 0.7	Vitamin D:2.6 ± 0.9 Se:2.7 ± 0.8 MI:2.7 ± 1.0 Placebo:3.1 ± 1.0
Robert Krysiak 2018-1	Vitamin D:100μg(4000IU);Selenomethyionine:200μg/d	Vitamin D:835 ± 245 Se:878 ± 286	Vitamin D:638 ± 211 Se:649 ± 226	Vitamin D:756 ± 302 Se:783 ± 312	Vitamin D:562 ± 267 Se:570 ± 243	Vitamin D:2.9 ± 0.7 Se:2.8 ± 0.8	Vitamin D:2.7 ± 0.7 Se:2.6 ± 0.6
Robert Krysiak 2018-2	Selenium:200μg/d	Se:896 ± 295 Placebo:975 ± 324	Se:551 ± 206 Placebo:765 ± 287	Se:829 ± 302 Placebo:867 ± 368	Se:549 ± 184 Placebo:671 ± 211	Se:2.1 ± 0.9 Placebo:2.3 ± 1.0	Se:1.8 ± 0.8 Placebo:2.1 ± 0.9
Qu Chunmei 2018	Selenium:200μg/d	Se:1182.47 ± 128.26 Placebo:1165.43 ± 163.45	Se:791.5 ± 172.8 Placebo:1206.54 ± 165.45	Se:974.34 ± 100.95 Placebo:991.39 ± 108.08	Se:524.22 ± 103.92 Placebo:988.92 ± 96.75	Se:2.61 ± 1.18 Placebo:2.77 ± 1.09	Se:2.7 ± 0.91 Placebo:2.64 ± 1.29
Lv Xiaofei 2018 (36)	Selenium:200μg/d	Se:1208 ± 136 Placebo:1175 ± 155	Se:805 ± 175 Placebo:1237 ± 130	Se:984 ± 100 Placebo:970 ± 108	Se:525 ± 104 Placebo:1027 ± 111	Se:2.56 ± 1.24 Placebo:2.75 ± 1.30	Se:2.35 ± 1.95 Placebo:2.48 ± 1.8
Han Na 2018	Selenium:200μg/d	Se:349.23 ± 24.64 Placebo:348.73 ± 22.31	Se:188.98 ± 15.13 Placebo:377.1 ± 21.31	Se:287.66 ± 21.03 Placebo:280.39 ± 20.78	Se:131.64 ± 17.73 Placebo:279.1 ± 22.81	Se:6.64 ± 0.89 Placebo:6.75 ± 0.88	Se:4.68 ± 0.57 Placebo:6.8 ± 0.83
Gao Hongxia 2017 (38)	Selenium:200μg/d	Se:376 ± 123 Placebo:357 ± 141	Se:306 ± 129 Placebo:419 ± 133	Se:1231 ± 343 Placebo:1271 ± 351	Se:1057 ± 381 Placebo:1443 ± 397	Se:2.77 ± 1.5 Placebo:2.83 ± 1.31	Se:2.39 ± 1.49 Placebo:2.98 ± 1.41
Zhou Jingying 2018 (39)	Vitamin D	NM	NM	NM	NM	NM	NM
Fu Liping 2019 (40)	Vitamin D(Calcitriol):0.25μg/d	Vitamin D:649.78 ± 252.25 Placebo:615.69 ± 354.66	Vitamin D:408.65 ± 218.65 Placebo:648.18 ± 346.61	Vitamin D:213.49 ± 89.22 Placebo:218.13 ± 157.75	Vitamin D:133.72 ± 83.61 Placebo:230.43 ± 143.48	Vitamin D:2.84 ± 2.19 Placebo:2.52 ± 1.49	Vitamin D:2.48 ± 1.60 Placebo:2.94 ± 1.10

In 7 of these articles, interventions lasted 6 months, while in 3 articles, interventions lasted 3 months. Of these 10 studies, 1 was an open trial, which was considered of moderate quality, and the remaining studies were randomised controlled trials, all of high quality (**Table 2**).

### 3.2 Network meta-analysis plot

**Figure 1:**
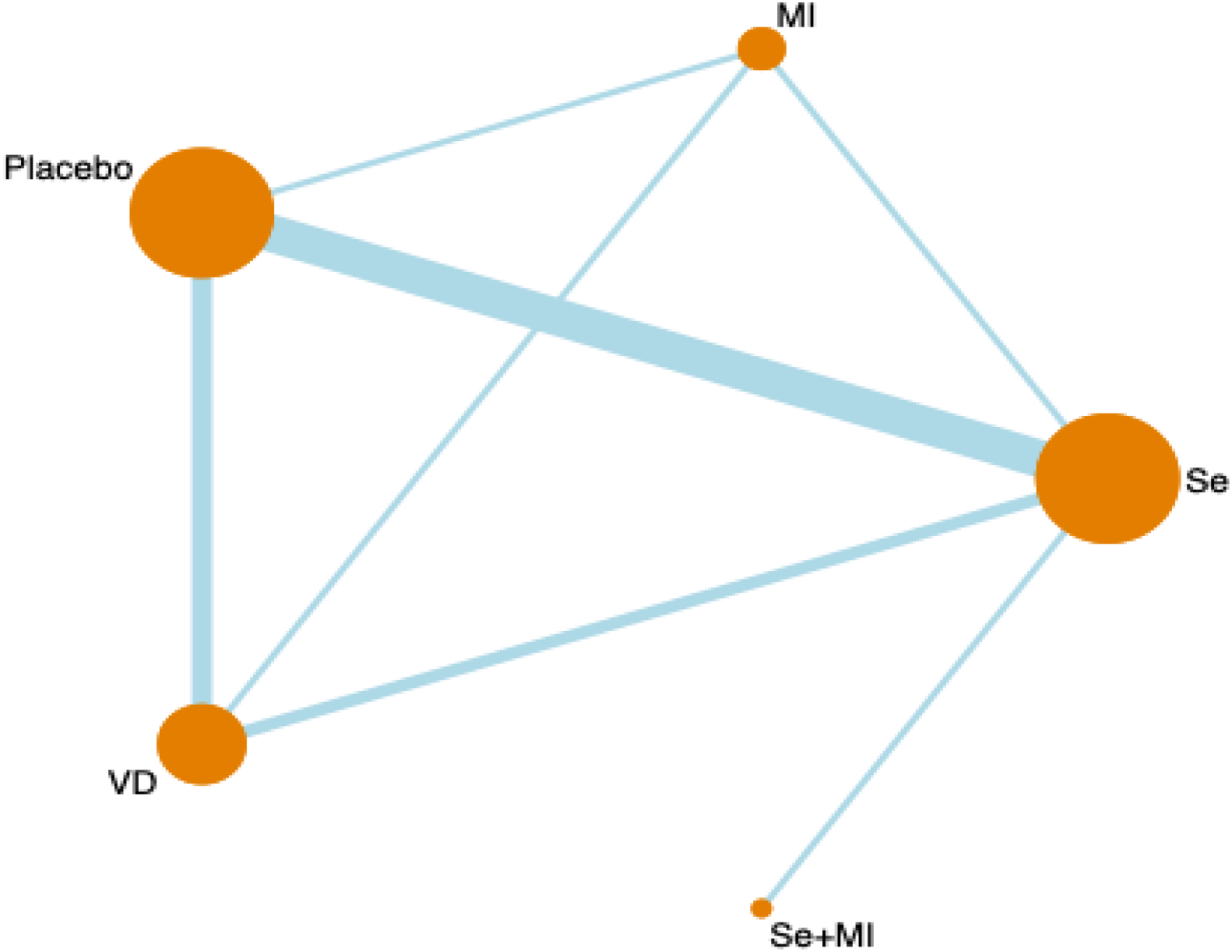
Network meta-analysis plot.

**Figure 1** shows a network diagram of trials that tested the effectiveness of different interventions (placebo, Vitamin D, selenium supplements, inositol, Vitamin D + selenium, Vitamin D + inositol) and direct comparisons of these interventions. Node size is the sample size for each type of intervention, and line thickness is proportional to the number of trials for that comparison.

### 3.3 Forest plots

**Figure 2:**
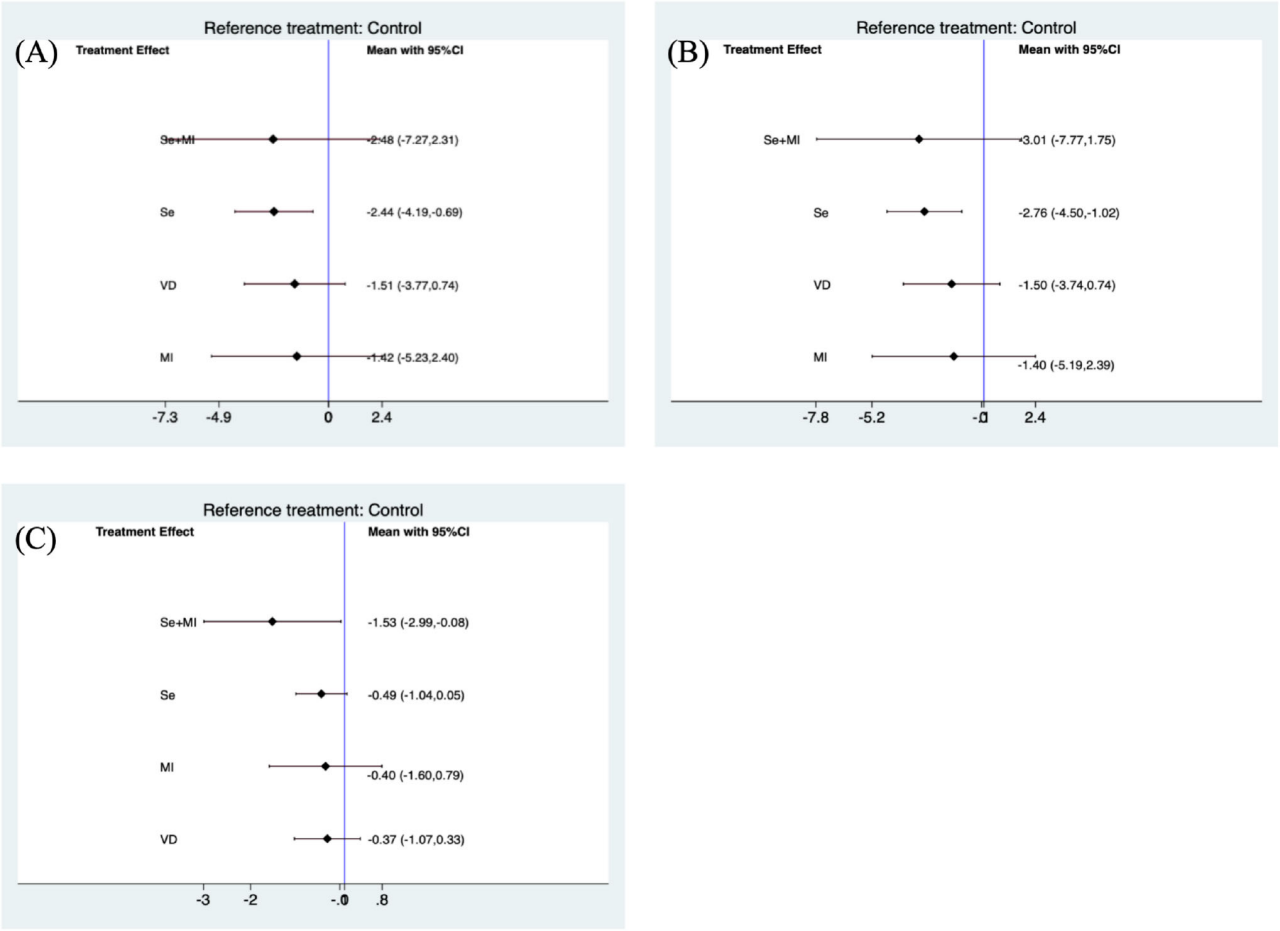
Forest plots of TPOAb and TgAb levels during supplement use. **(A)** Forest plot of TPOAb levels when using Se, VD, Se+MI and MI supplements vs. placebo. **(B)** Forest plot of TgAb levels when using Se, VD, Se+MI and MI supplements vs. placebo. **(C)** Forest plot of TSH levels when using Se, VD, Se+MI and Myo-inositol supplements vs. placebo. Se, Selenium; MI, Myo-inositol; VD, Vitamin D.

The use of selenium supplementation was generally able to significantly reduce TPOAb (SMD(Standard mean difference): -2.44, 95% CI(Confidential intervals):-4.19, -0.69) (**Figure 2A**) and TgAb(SMD: -2.76, 95% CI: -4.50, -1.02) (**Figure 2B**) levels when compared to the placebo group. The combination of selenium and Myo-inositol supplementation was able to reduce individual autoantibody levels when compared to selenium alone. However these results were not statistically significant in the meta-analysis, which is likely attributable to the limited sample size. In clinical trials, vitamin D may reduce antibody titres in HT patients, but this was not reflected in this study (**Figures 2A, B**). In clinical trials, the majority of HT patients who received vitamin D supplementation exhibited significant hypothyroidism. However, the sample size was limited, which raises concerns about the reliability of the findings. While inositol alone may not significantly reduce antibody titres, its combination with selenium or vitamin D may enhance the function of these supplements. Nevertheless, further research is needed to clarify its role in clinical practice.

Due to inconsistencies in the treatment duration of the included trials, a separate subgroup analysis was conducted for trials that included treatment supplementation for a period of 6 months. The use of selenium supplements was still found to be effective in reducing TPOAb(SMD: -2.98, 95 %CI: -5.40, -0.55) and TgAb levels (SMD: -3.40, 95%CI: -5.67, -1.13).

**Figure 3:**
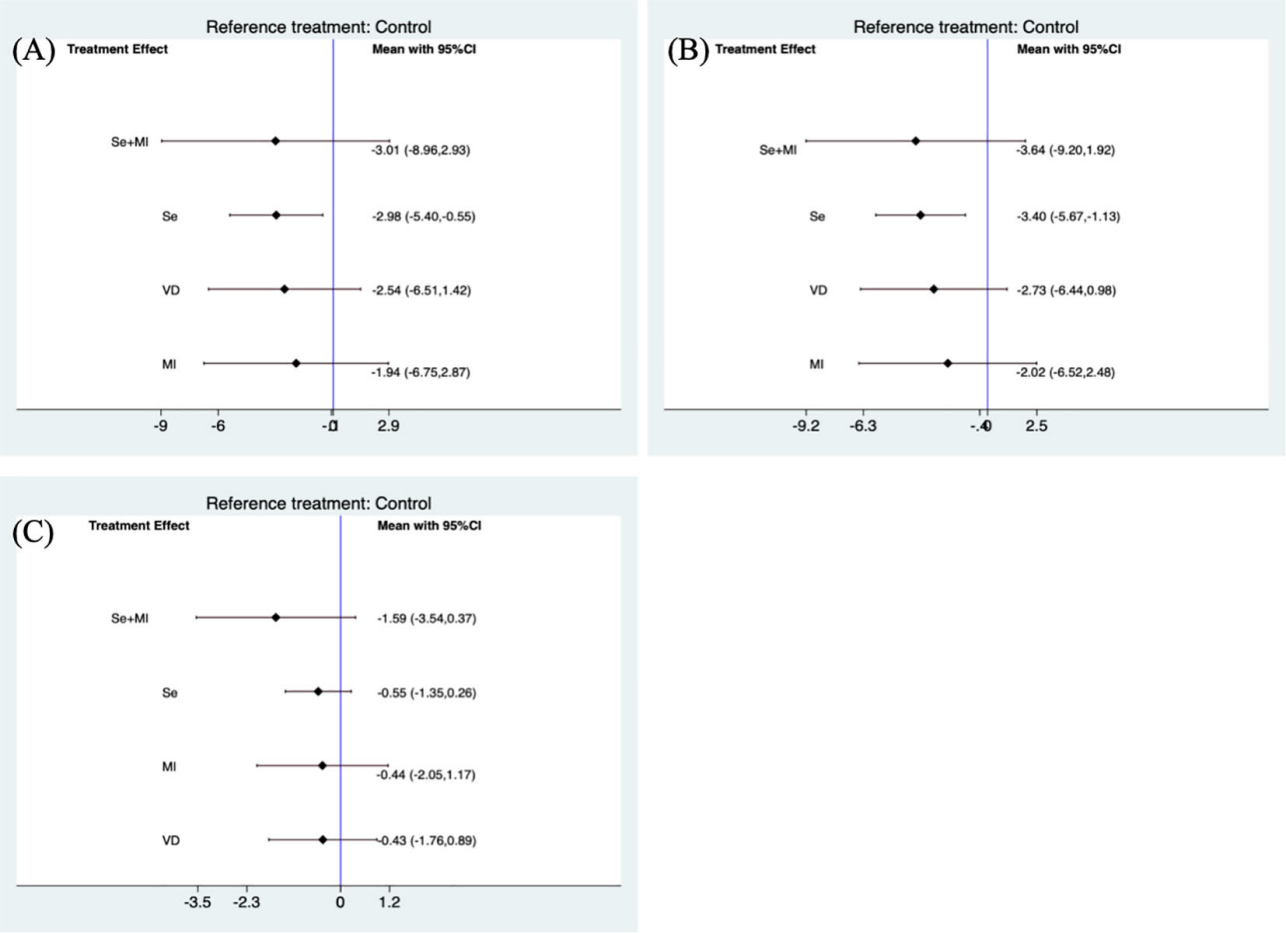
Forest plot of TPOAb and TgAb levels during supplement use for 6 months.

Furthermore, Myo-inositol used in combination with selenium was able to reduce TSH levels (SMD: -1.53, 95%CI: -2.99, -0.08) (**Figure 2C**). However these results were not significant when subgroup analyses were performed for studies using treatment supplements for 6 months (**Figure 3C**).

### 3.4 Publication bias

Because many of the articles on selenium supplementation came from CNKI, we conducted a publication bias test on the included articles. The results of Egger’s test indicated the presence of publication bias, and the results did not reverse after using the trim and fill method, indicating that the results are stable and publication bias does not affect this result.

### 3.5 Sensitivity analysis

To assess the stability of the results, we performed a sensitivity analysis. Each article was successively excluded and a meta-analysis was performed on the remaining literature, and we found that the results did not change significantly, which means that the results are stable and reasonable.

## 4 Discussion

In this network meta-analysis, selenium supplementation during the treatment of HT patients effectively reduced TPOAb (SMD:-2.44, 95% CI:-4.19, -0.69) and TgAb (SMD: -2.76, 95% CI:-4.50, -1.02) levels. If patients have vitamin D deficiency or insufficiency(<20 ng/mL) or low selenium levels(20-30 ng/mL) (41) and still have high autoantibodies after long-term regular treatment, we can consider adding selenium or vitamin D or a combination of the two, or inositol or a combination of the two. If a single supplement does not significantly reduce antibody levels, a combination of supplements is more likely to be recommended. Furthermore, it is recommended that patient levels of selenium and vitamin D be monitored throughout the course of treatment, with adjustments to supplement dosages made as necessary.

The association between autoimmune thyroid disease and low levels of vitamin D has been confirmed by many epidemiological studies (42, 43), with several investigations showing a negative correlation between vitamin D levels and thyroid-associated antibodies (44, 45). Consequently, researchers have investigated the potential of vitamin D supplementation to improve autoimmune thyroiditis and reduce the associated autoantibody levels. While some studies have found that vitamin D supplementation in HT patients treated with levothyroxine can reduce autoantibody levels, it seems to be more effective for patients with low vitamin D levels (34, 46, 47). It is undeniable that vitamin D may be involved in the pathogenesis of HT. Recent studies have also found that vitamin D supplementation under normal conditions is an effective method in reducing the prevalence of autoimmune diseases (48).

There is a growing body of evidence to identify that selenium plays a role in thyroid function (49), and recent studies have shown that selenium levels are negatively correlated with autoantibody titres in patients with HT (50). Therefore, clinicians tend to favour the use of selenium supplements as an adjunctive treatment HT (51). Furthermore, oxidative stress has been suggested to be involved in the pathogenesis of HT (52). Glutathione peroxidase is a crucial antioxidant enzyme in humans, and selenium acts as its catalytic centre during reduction reactions (53). Moreover, selenium has been demonstrated to inhibit inflammatory responses by modulating the ratio of Treg/Th17 cells(Regulatory T-cells/T-helper cell 17) (54). Our results are in agreement with studies reporting that selenium supplementation significantly reduces thyroid-associated autoantibody levels. However, that excessive selenium intake can result in toxicity (55). Therefore, supplementation should be moderate and tailored to the individual.

Myo-inositol is a vital nutrient for human health and an essential component of cellular structural lipids (56). Derivatives of Myo-inositol perform a number of important cellular and metabolic functions. including morphogenesis (57), cytoskeletal rearrangement (58), cell proliferation (59), and the regulation of glucose metabolism (60). In the thyroid gland, an imbalance in Myo-inositol metabolism impairs the hormone biosynthesis, storage, and secretion (61). In recent years, Myo-inositol has been effectively used in combination with selenium or vitamin D as a dietary supplement for the treatment of HT. This combination can adequately reduce autoantibody levels and restore TSH levels, while also restoring TSH signalling (31, 62, 63). Nevertheless, further clinical trials and mechanistic studies are required to provide additional support for these findings.

It is important to note that while the use of Myo-inositol in clinical practice is increasing, Myo-inositol alone is not as effective as when combined with selenium or vitamin D (31). A study by Payer found that combining inositol and selenium in women with subclinical hypothyroidism resulted in a reduction in autoantibody titres and a decline in serum cholesterol levels in patients (62). Krysiak et al. observed that the impact of vitamin D on thyroid autoimmunity and hypothalamic-pituitary-thyroid axis activity was more pronounced in patients with HT who received Myo-inositol (64). Furthermore, Benvenga et al. administered selenium and Myo-inositol to mice with cadmium-induced thyroid damage and observed that mice treated with both inositol and selenium exhibited the same indices as those not treated with cadmium. These findings indicate that the combination of Myo-inositol and selenium protects the thyroid in cadmium-exposed subjects (65). Moreover, the effect of selenium combined with vitamin D on the reduction thyroid-specific autoantibodies should not be overlooked. Krysiak et al. administered vitamin D (4000 IU/day) to 23 women who had been treated with selenomethionine for a minimum of 12 months. The combination of vitamin D and selenothionine was found to be effective in lowering TPOAb and TgAb titres and increasing SPINA (The secretory capacity of the thyroid gland), when compared to women treated with vitamin D alone (24 women). This indicates that selenium intake may enhance the effect of vitamin D in thyroid autoimmunity (33). The combination of multiple supplements is likely to become a future trend in clinical practive for the reduction of HT-specific autoantibody titres.

To our knowledge, this is the meta-analysis to compare the efficacy of divers dietary supplements as an adjunctive therapy for the management of HT. Furthermore, patients treated with levothyroxine were excluded from this analysis, avoiding drug influences on the assessed measures. However, this resulted in some limitations, including a stendency towards a higher number of included selenium-related articles. Conversely, Myo-inositol, which has been proposed as a dietary supplement for HT patients in recent years, has fewer reported clinical studies. In addition, discrepancies in treatment duration, variations in drug dosage, and differences in drugs administration may also influence the reported experimental outcomes. Nevertheless, this meta-analysis revealed a statistically significant reduction in autoantitody titres when selenium was administered. Further research is required to investigate the efficacy of other supplements in this clinical setting.

## 5 Conclusion

In conclusion, the results of this study demonstrated that selenium supplementation has a significant role in lowering thyroid autoantibody titres in patients with HT. Moreover, large multicentre randomised controlled studies are required to ascertain whether other supplement-assisted treatments for HT can prove beneficial for these patients.
